# ADAM17-mediated CD44 cleavage promotes orasphere formation or stemness and tumorigenesis in HNSCC

**DOI:** 10.1002/cam4.147

**Published:** 2013-10-16

**Authors:** Pachiyappan Kamarajan, Jae M Shin, Xu Qian, Bibiana Matte, Joey Yizhou Zhu, Yvonne L Kapila

**Affiliations:** Department of Periodontics and Oral Medicine, University of Michigan School of DentistryAnn Arbor, Michigan

**Keywords:** ADAM17, CD44, HNSCC, MMPs, oral cancer, orasphere, stemness, tumorigenesis

## Abstract

CD44, an extracellular matrix (ECM) receptor, has been described as a cancer stem cell marker in multiple cancers, including head and neck squamous cell carcinoma (HNSCC). HNSCC orasphere formation or stemness was characterized by cleavage of CD44, and thus we hypothesized that this proteolytic processing may be critical to stemness and tumorigenesis. We tested this hypothesis by examining the mechanisms that regulate this process in vitro and in vivo, and by exploring its clinical relevance in human specimens. Sphere assays have been used to evaluate stemness in vitro. Spheres comprised of HNSCC cells or oraspheres and an oral cancer mouse model were used to examine the significance of CD44 cleavage using stable suppression and inhibition approaches. These mechanisms were also examined in HNSCC specimens. Oraspheres exhibited increased levels of CD44 cleavage compared to their adherent counterparts. Given that disintegrin and metalloproteinase domain-containing protein 17 (ADAM17) is a major matrix metalloproteinase known to cleave CD44, we chemically inhibited and stably suppressed ADAM17 expression in HNSCC cells and found that these treatments blocked CD44 cleavage and abrogated orasphere formation. Furthermore, stable suppression of ADAM17 in HNSCC cells also diminished tumorigenesis in an oral cancer mouse model. Consistently, stable suppression of CD44 in HNSCC cells abrogated orasphere formation and inhibited tumorigenesis in vivo. The clinical relevance of these findings was confirmed in matched primary and metastatic human HNSCC specimens, which exhibited increased levels of ADAM17 expression and concomitant CD44 cleavage compared to controls. CD44 cleavage by ADAM17 is critical to orasphere formation or stemness and HNSCC tumorigenesis.

## Introduction

Oral cancer is one of the leading causes of death worldwide, and oral squamous cell carcinoma (OSCC) accounts for more than 90% of oral malignancies [1]. OSCC, a subtype of head and neck squamous cell carcinoma (HNSCC) has a poor 5-year survival rate that has not improved in decades. This underscores the need to further examine its pathogenesis and to identify novel biomarkers and new therapeutic approaches to treat oral cancer. Although, smoking, alcohol consumption, and human papillomavirus (HPV) infection are known risk factors for oral cancer, other mechanisms that contribute to its pathogenesis, such as the role of cancer stem cells, are not completely understood, yet merit further investigation and may possess significant therapeutic promise.

CD44, a cell surface glycoprotein that functions as a receptor for hyaluronic acid (HA), a component of the extracellular matrix (ECM), has been identified as a cancer stem cell marker in many cancer types, including breast, brain, prostate, and HNSCC [2–5]. In HNSCC, a significant association was found between CD44 expression and poor 5-year survival rates in patients with SCC of the oro- and hypopharynx, and larynx [6]. CD44 mediates multiple effects on cell behavior in part through its ability to interact with HA [7] and due to the presence of multiple isoforms of CD44 that arise from N- and O-glycosylation, alternative splicing, and proteolytic cleavage. These multiple forms of CD44 may help explain how this fairly ubiquitous protein can mediate such an array of interactions [8]. These interactions can affect cell adhesion [4, 5, 9], cell proliferation [10], cell motility, cell survival [11], cell migration [12], tumor metastasis [13], chemoresistance [14], and apoptotic and anoikis resistance [8, 15–17]. Anoikis is a form of apoptotic cell death triggered by loss of ECM contacts, and anoikis resistance is a form of survival and anchorage-independent growth that contributes to tumorigenesis [18–20], including HNSCC [21–25]. Anchorage-independent growth leads to sphere formation and these assays have been used to evaluate stemness in vitro [3, 26]. Indeed, these sphere assays have been used to identify and study cancer stem cells in HNSCC [27–29]. These studies suggest that CD44 plays an important role in HNSCC tumor initiation by imparting cells with enhanced anoikis-resistance properties and/or stemness.

CD44 can undergo proteolytic cleavage or shedding by a variety of proteases. This cleavage also leads to the release of its cytoplasmic domain from the membrane into the cytoplasm. This, in turn, perturbs the interaction between CD44 and attached protein complexes of the cytoskeleton and growth factor signaling cascades. Additionally, the cytoplasmic domain can enter the nucleus and act as a transcription factor, changing the gene expression pattern of cells [30, 31]. Furthermore, shedding of the ectodomain of CD44 plays a critical role in tumor cell migration [12, 31]. Several proteases are involved in the proteolytic processing of CD44, including proteins containing a disintegrin and metalloprotease domain; namely ADAMs and membrane-type metalloproteases (MT-MMPs). ADAMs are a family of cell-surface proteases that are related to MMPs and contain several functional domains, including a metalloproteinase, disintegrin, cysteine-rich, and transmembrane domain, as well as a cytoplasmic tail. Cleavage of CD44 via proteases, including disintegrin and metalloproteinase domain-containing protein 17 (ADAM17), is associated with metastasis in OSCC [32, 33]. Thus, the shedding of CD44 can have important effects on tumor cell behavior; however, its effects on orasphere formation or stemness have not been explored. Identification of key mediators of this process could be used to identify therapeutic targets for the treatment of HNSCC. In this study, we examined the role of CD44 cleavage in the context of HNSCC orasphere formation or stemness and tumorigenesis. Our data demonstrate, for the first time that CD44 cleavage by ADAM17 promotes stemness and tumorigenesis in HNSCC.

## Material and Methods

### Institutional review board approval

This study has been approved by and was conducted in accordance with the regulations set for by the institutional review board and the committee on the use and care of animals at the University of Michigan.

### Cell lines and culture

Head and neck squamous cell carcinoma cells (UM-SCC-14A, a gift from Tom Carey, University of Michigan and HSC-3, a gift from Randy Kramer, University of California-San Francisco) were maintained in Dulbecco's modified Eagle's medium containing 10% fetal bovine serum, 1% penicillin, and 1% streptomycin in a 5% CO_2_ atmosphere at 37°C.

### Oraspheres and control adherent HNSCC cells

Anoikis-resistant oraspheres and adherent control HNSCC (UM-SCC-14A and HSC-3) cells were prepared as previously reported [19, 24, 25]. These HNSCC oraspheres were developed by maintaining cells under suspension conditions on poly-HEMA coated plates (7.5 mg/mL in 95% ethanol, Sigma-Aldrich, St. Louis, MO) for 6 days. In this assay, cells that survive anchorage withdrawal form multicellular aggregates or oraspheres [24, 25]. The TAPI2 (ADAM17) inhibitor (EMD Millipore, Billerica, MA) was reconstituted in water and used in the orasphere assays. G1254023X (ADAM10 inhibitor) and GW280264X (ADAM10 and ADAM17 inhibitor) were gifts from Dr. Andreas Ludwig (Christian-Albrechts-University, Kiel, Germany).

### Immunoblot analysis

To evaluate the expression of CD44 and ADAM17 in cell lysates, cells were washed once with phosphate-buffered saline and lysed on ice for 30 min in RIPA lysis buffer (R0278; Sigma-Aldrich) containing 1% protease inhibitor cocktail (P8340; Sigma-Aldrich). For subcellular localization experiments, cytosolic and nuclear fractions from cells were prepared using the NE-PER nuclear and cytoplasmic extraction kit (Pierce, Rockford, IL). To evaluate the expression levels of CD44 and ADAM17 in tissues, frozen human HNSCC tissue specimens were obtained (Proteogenex Inc., Culver City, CA) and analyzed. Tissue specimens were histologically confirmed to contain over 90% tumor tissue prior to analysis. All tissues were snap frozen in liquid nitrogen immediately after surgery and preserved at −80°C. A total of 18 tissues from six patients (matched normal adjacent tissue, primary tumors, and metastatic tumors from within the same patients) were investigated in this study. Tissue specimens were minced and homogenized in RIPA lysis buffer (R0278; Sigma-Aldrich) containing 1% protease inhibitor cocktail (P8340; Sigma-Aldrich) and centrifuged at 15,000*g* to remove insoluble material. Lysates were adjusted for protein concentration with the bicinchoninic acid (BCA) protein assay kit (Bio-Rad, Hercules, CA), resolved by sodiumdodecyl sulfate polyacrylamide gel electrophoresis (SDS-PAGE) and transferred to Immobilon-P membranes (Millipore). Blots were probed with a CD44 (SC-7946; Santa Cruz Biotechnology, Santa Cruz, CA), ADAM17 (SC-13973; Santa Cruz Biotechnology), GAPDH (CS204254; Millipore) or Histone H3 (05-1341; Millipore) primary antibody, followed by a horseradish peroxidase-conjugated anti-rabbit antibody (SC-2004; Santa Cruz Biotechnology), then developed with the ECL-Plus detection system (Pierce). To demonstrate equal protein loading, membranes were stripped and reprobed with an anti-β-actin antibody (SC-1615; Santa Cruz Biotechnology). All other reagents were from Sigma.

### Development of stable cell lines

UM-SCC-14A cells were transduced with ADAM17-shRNA (SC-36604-V), CD44-shRNA (SC-29342-V), or scrambled-shRNA (SC-108080; Santa Cruz Biotechnology) lentiviral particles in 0.5 mL of serum-free media, and then selected in 10 μg/mL puromycin (sc-108071; Santa Cruz Biotechnology) for an additional 10 days. Surviving cell colonies were picked and propagated before testing for ADAM17 and CD44 expression using Western blot analysis.

### Immunodeficient oral cancer mouse xenograft model

To validate the significance of ADAM17 and CD44 in regulating orasphere formation or stemness and tumorigenesis in vivo, HNSCC cells that exhibited stably suppressed levels of ADAM17 or CD44 and control transduced cells were tested in an oral cancer mouse model as described earlier [25, 34, 35]. For these experiments, HNSCC cells with and without altered ADAM17 and CD44 levels were suspended in DMEM, chilled on ice, and resuspended in an equal volume of growth factor reduced Matrigel (BD Biosciences, San Jose, CA) to a final concentration of 1 × 10^6^ cells/0.05 mL prior to injection. A total volume of 0.05 mL was injected sub-mucosally into the floor of the mouth. Six weeks after injection, mice were euthanized and tumor incidence and/or volume were evaluated as previously described [25, 34, 35].

### Statistical analysis

In general, values are expressed as means ± SD. Intergroup differences were determined by two-way analysis of variance and Scheffe's multiple-comparison test. Statistical significance was defined as *P* ≤ 0.05. All experiments were repeated at least three times. For the in vivo studies, independent *t*-tests with unequal variances were used.

## Results

### CD44 cleavage is a signature of orasphere formation or stemness

**Figure 1:**
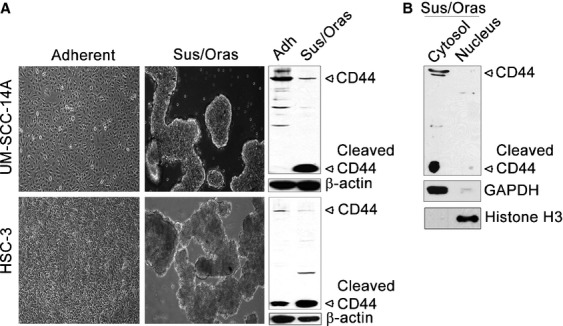
Head and neck squamous cell carcinoma (HNSCC) cell oraspheres in suspension exhibit greater levels of CD44 cleavage. (A) (left) Phase-contrast images of UM-SCC14A and HSC-3 cells under adherent and suspension conditions (Sus/Oras) and (right) immunolots showing the levels of CD44 expression in UM-SCC14A and HSC-3 cells in adherent (Adh) and suspension conditions (Sus/Oras). (B) Immunolot showing the levels of CD44 expression in cytosolic and nuclear fractions in UM-SCC14A cells cultured under suspension conditions. GAPDH and Histone H3 served as cytosolic and nuclear markers, respectively. β-actin served as loading control.

To determine the role of CD44 in orasphere formation or stemness, we examined the expression of CD44 in oraspheres and control/adherent HNSCC cells (UM-SCC-14A and HSC-3). As reported in our previous publication [24, 25], when HNSCC cells become anoikis resistant they form multicellular aggregates or oraspheres in suspension conditions. This ability to form spheres in suspension is used as a measure of stemness. We found that as HNSCC cells formed oraspheres they exhibited CD44 cleavage (Fig. 1A). This proteolytic processing of CD44 led to high levels of a small molecular weight fragment of ∼25 kDa that was identifiable by Western blotting. Adherent control cells did not reveal comparable cleavage. Given that cleavage products of CD44 can localize to the nucleus and mediate signaling events, we investigated the subcellular localization of the CD44 cleaved fragments by Western blotting. We found that the major cleaved fragment of CD44 (∼25 kDa) is exclusively found in the cytosolic fraction (Fig. 1B).

### ADAM17 mediates cleavage of CD44 and thereby promotes orasphere formation or stemness

**Figure 2:**
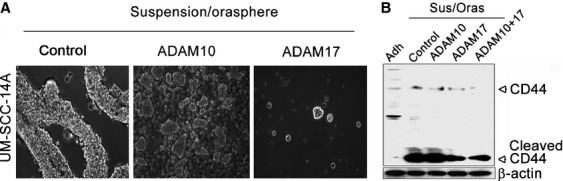
Inhibiting CD44 cleavage with a chemical inhibitor of ADAM17 (TAPI2) abrogates orasphere formation in UM-SCC14A cells. (A) Phase-contrast images of UM-SCC 14A cells cultured under suspension conditions. Cells were either untreated (control) or pretreated with an ADAM10 (G1254023X) or ADAM17 (TAPI2) chemical inhibitor for 2 h and continuously cultured under suspension conditions for 1 day. (B) Immunoblot showing the levels of CD44 expression in untreated control cells (under adherent or suspension conditions) and cells treated with inhibitors to ADAM10 (G1254023X), ADAM17 (TAPI2), or ADAM10 and 17 (GW280264X) under suspensions conditions.

**Figure 3:**
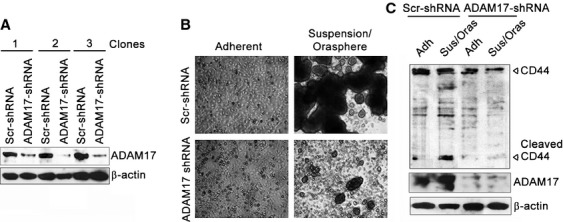
ADAM17 suppression blocks orasphere formation and inhibits CD44 cleavage in UM-SCC14A cells. (A) Immunoblots showing the levels of ADAM17 expression in cells transduced with scr-shRNA or ADAM17-shRNA. Three different clones are shown. (B) Phase-contrast images of cells stably transduced with scr-shRNA or ADAM17-shRNA then cultured under adherent or suspension conditions (Orasphere) for 6 days. (C) Immunoblots showing the levels of CD44 and ADAM17 expression in cells transduced with scr-shRNA or ADAM17-shRNA and grown under adherent and suspension conditions as in B.

To specifically explore the role of CD44 cleavage in the process of orasphere formation, we inhibited major matrix metalloproteinases responsible for CD44 cleavage, namely ADAM10 and ADAM17. An ADAM17 inhibitor (TAPI2), inhibited CD44 cleavage in HNSCC cells and prevented orasphere formation (Fig. 2A and B), supporting the concept that ADAM17-mediated cleavage of CD44 regulates HNSCC cell stemness. However, an ADAM10 inhibitor (G1254023X) exhibited lesser effects on orasphere formation and cleavage of CD44. In addition, stable suppression of ADAM17 in HNSCC cells once again inhibited CD44 cleavage and prevented orasphere formation or stemness (Fig. 3A–C). In contrast, control transduced cells in suspension conditions continued to show pronounced cleavage of CD44 and orasphere formation.

### Stable suppression of ADAM17 in HNSCC cells reduces tumor burden in vivo

**Figure 4:**
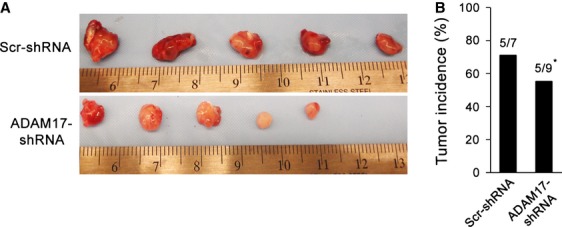
ADAM17 suppression reduces tumor burden in vivo. (A) Images show the dissected tumors and (B) percentage of tumor incidence in mice that were injected with UM-SCC-14A cells that had been transduced with scrambled shRNA or ADAM17 shRNA and grown under adherent conditions (*P ≤ 0.05).

To confirm the importance of ADAM17 in promoting stemness and thereby tumorigenesis in vivo, HNSCC cells with stably suppressed levels of ADAM17 or control transduced cells, both grown under adherent conditions, were injected into a floor-of-mouth oral cancer mouse model. Injection of HNSCC cells with stably suppressed levels of ADAM17 led to significantly reduced tumor volumes and incidence (Fig. 4A and B; Table 1). In contrast, injection of control transduced cells led to higher tumor volumes and incidence. As shown in the graph in Figure 4, only 5/9 (56%) mice developed tumors when injected with HNSCC cells that exhibited stable suppression of ADAM17, whereas 5/7 (71%) mice developed tumors when injected with HNSCC cells derived from control transduced cells expressing normal levels of ADAM17. Of the tumors that did develop in mice injected with the HNSCC cells with stably suppressed levels of ADAM17, the mean tumor volume was only 29.8 mm^3^, whereas the tumors in mice injected with control transduced cells reached a mean tumor volume of 117.8 mm^3^. Thus, ADAM17 is a critical determinant of HNSCC tumorigenesis.

**Table 1:** Tumor volumes for mice injected with UM-SCC-14A cells

Animal	Scr-shRNA	ADAM17-shRNA
	
	Tumor volume (mm^3^)	Tumor volume (mm^3^)
1	101	64
2	137	32
3	136	20
4	127	17
5	88	16
6	No tumor	No tumor
7	No tumor	No tumor
8		No tumor
9		No tumor
Mean volume	**117.8±19**	**29.8**±18*

Statistical analysis: Independent *t*-test with unequal variances

* *P*≤ 0.01.

### CD44 downregulation inhibits orasphere formation or stemness in vitro and tumorigenesis in vivo

**Figure 5:**
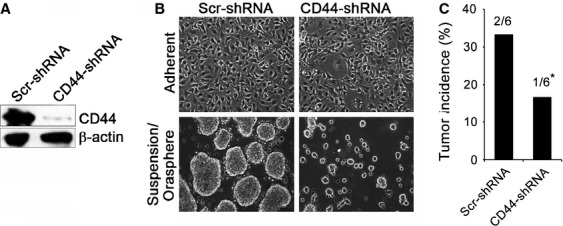
CD44 suppression inhibits orasphere formation in vitro and reduces tumor incidence in vivo. (A) Immunoblots showing the levels of CD44 in cells transduced with scrambled shRNA or CD44-shRNA. (B) Phase-contrast images of cells stably transduced with scr-shRNA or CD44-shRNA then cultured under adherent or suspension conditions (Orasphere) for 6 days. (C) Percentage of tumor incidence in mice that were injected with UM-SCC-14A cells transduced with scrambled shRNA controls or CD44 shRNA and grown under adherent conditions (*P ≤ 0.05).

It stands to reason that while inhibiting cleavage of CD44 can inhibit orasphere formation, loss of CD44 would similarly impair this process. Thus, to further elucidate the importance of CD44 in mediating stemness in HNSCC, we performed orasphere assays with HNSCC cells exhibiting stably suppressed levels of CD44. As expected, stable suppression of CD44 expression in HNSCC cells inhibited orasphere formation (Fig. 5A and B), underscoring the important role of CD44 in this process. Similarly, we surmised that suppressing stemness by stably suppressing CD44 would also impair tumorigenesis. Thus, to further confirm the role of CD44 in modulating stemness and HNSCC tumorigenesis in vivo, we again used the murine floor-of-mouth model that mimics human HNSCC. In agreement with in vitro findings, our mouse model data showed that suppression of CD44 in HNSCC cells significantly reduced tumor incidence in vivo (Fig. 5C). As shown in the graph in Figure 5C, only 1/6 (17%) of mice developed tumors when injected with HNSCC cells that exhibited stable suppression of CD44 (adherent), whereas 2/6 (33%) of mice developed tumors when injected with HNSCC cells expressing normal levels of CD44 (adherent). Thus, these data further confirm the importance of CD44 in stemness and tumorigenesis, and that oraspheres and the stemness phenotype within, is an important functional feature underlying tumorigenesis.

### ADAM17 expression and CD44 cleavage are concomitantly elevated in human HNSCC tissue specimens

**Figure 6:**
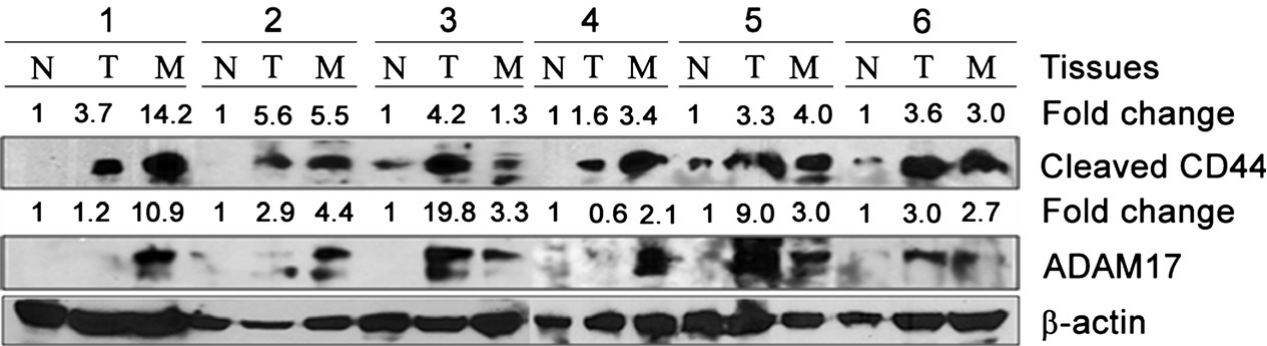
ADAM17 expression and CD44 cleavage are concomitantly elevated in human head and neck squamous cell carcinoma (HNSCC) tissue specimens. Immunoblots showing the levels of CD44 cleavage and ADAM17 expression in HNSCC tissue specimens (N, normal adjacent tissue; T, primary tumor; M, metastatic tissue), normalized to β-actin. Fold change in CD44 and ADAM17 expression in primary and metastatic tumors relative to normal adjacent tissues was assessed using densitometric analysis.

The clinical relevance of these findings was examined in matched sets of human HNSCC tissue specimens. Specifically, we examined normal adjacent tissue, primary tumors, and metastatic tumors from within the same patients. The clinical characteristics of the specimens are reported in Table 2. The tumor specimens were all advanced stage IV tumors, with a T2–T4 and N1–N2 profile and metastatic lesions to the lymph nodes. The majority of the tumors exhibited a poorly differentiated histological grade 3 profile. The data showed that the primary and metastatic tumor samples exhibited increased levels of ADAM17 expression and concomitant CD44 cleavage compared to normal controls (Fig. 6). These data support the concept that ADAM17-mediated cleavage of CD44 is an important determinant of the stemness phenotype that promotes HNSCC tumorigenesis.

**Table 2:** Clinical characteristics of HNSCC patients

Patient number	Sex/Age	Specimen type	Diagnosis (tissues)	Grade	TNM	Stage	Mets
1	M/59	Tongue/Lymph nodes	NAT/Tumor/Mets	G3	T4aN1M0	IVA	1/9
2	M/63	Laryngopharynx/Lymph nodes	NAT/Tumor/Mets	G3	T3N2M0	IVA	9/9
3	M/56	Larynx/Lymph nodes	NAT/Tumor/Mets	G3	T2N2M0	IVA	5/11
4	M/61	Larynx/Lymph nodes	NAT/Tumor/Mets	G2	T3N2cM0	IVA	5/19
5	M/60	Hypopharynx/Lymph nodes	NAT/Tumor/Mets	G3	T3N2bM0	IVA	3/7
6	M/54	Larynx/Lymph nodes	NAT/Tumor/Mets	G1	T3N2M0	IVA	12/37

## Discussion

Sphere assays have been used to measure anchorage-independent growth, anoikis resistance, and recently stemness [27–29, 36], and thus these processes can be considered somewhat synonymous. Interestingly, although sphere assays and the processes of anchorage-independent growth and anoikis resistance have been studied for a long time in the context of tumorigenesis [18] and the ECM, it is only recently that this property of growing tumor cells in low attachment conditions or three-dimensional matrices has been rediscovered and used to evaluate stemness. Indeed as early as 1974, it was recognized that anchorage-independent growth in vitro in the form of spherical colonies in semisolid medium can predict neoplastic growth in nude mice [37]. Also, other early studies with a focus on the role of the ECM have provided significant understanding about the properties regulating these sphere assays. Work by Kantak et al. [19] demonstrated that E-cadherin expression is critical to HNSCC sphere formation. Similarly, work by Zhang and colleagues, showed that a fibronectin ECM provides focal adhesion kinase (FAK)-mediated survival signals that promote HNSCC sphere formation [24]. Thus, a discussion on stemness as evaluated by sphere assays by necessity should incorporate studies and findings relevant to anchorage-independent growth and anoikis resistance of tumor cells.

With this in mind, a brief discussion on anoikis resistance as pertains to tumorigenesis is presented. Cancer cells that acquire malignant potential have developed mechanisms to resist anoikis, thereby allowing survival of cancer cells in systemic circulation and facilitating secondary tumor formation in distant organ sites [38]. Anoikis resistance is implicated in different human malignancies, including ovarian [39], breast [40], colon [41], and oral cancer [21]. Several signaling pathways have been reported to regulate anoikis, whereby involvement of integrin receptors, death receptors, adhesion molecules, and complex signaling cascades can lead to anoikis resistance and spread of metastatic cancer cells [22, 23, 42–45].

CD44 is a multifunctional protein involved in cell adhesion, migration, apoptosis resistance, drug resistance, and recently stemness. CD44 is a primary receptor for HA, a major component of the ECM, and it plays a critical role in cell signaling and cell-ECM interactions in cancer [46–48]. In fact, expression of various CD44 protein variants correlates with aggressive human cancers, including HNSCC and breast cancer [49]. Recent studies showed that CD44 overexpression serves as a prognostic factor in HNSCC and it is also associated with poor prognosis in HNSCC [6, 50–52]. Importantly, it has been proposed that cancer-initiating cells (cancer stem cells) constitute a minor population of cells within a tumor, and these stem cells can initiate the formation of new tumors [6, 53] and possess the capacity to metastasize [54]. These cells, including those in HNSCC, are enriched with CD44 expression [5, 55, 56]. Thus, CD44 is believed to be a stem cell marker in head and neck cancer as in other cancers [15, 57]. Our findings provide new evidence that CD44 cleavage plays an important role in mediating stemness in HNSCC. Our initial finding that CD44 cleavage was increased in HNSCC oraspheres compared to the control/adherent HNSCC cell counterparts led us to hypothesize that CD44 cleavage may play an important role in regulating stemness. Specifically, cleavage of CD44 could promote disruption of cell-ECM contacts and promote cell–cell contacts that drive sphere formation. Thus, to further explore the relevance of increased CD44 cleavage in the process of sphere formation, we used an inhibitor to ADAM17, a major protease known to cleave CD44. This inhibitor has been used to inhibit CD44 cleavage in several types of cancer [32]. Furthermore, ADAM17 itself has been implicated in regulating tumorigenesis via mechanisms that include targeting and inhibition of a Sox9/ADAM17 Axis by miR145. This mechanism also seems to involve release/activation of IL-6 and epidermal growth factor receptor (EGFR)-phosphoinositide 3-kinase (PI3K/AKT) [58, 59]. In agreement with these studies, inhibiting ADAM17 activity or expression also blocked HNSCC orasphere formation in our in vitro assays and tumorigenesis in our in vivo model. These findings indicate that CD44 cleavage by ADAM17 is critical to the process of stemness and HNSCC tumorigenesis.

The specific cleavage or shedding of CD44 into several fragments and their biological significance has been the focus of several studies. In a review by Nagano and Saya [31], several important and relevant topics were discussed including the fact that ectodomain cleavage of CD44 is highly prevalent in human tumors, that the soluble form of CD44 affects the function of membrane-bound CD44, that ectodomain cleavage of CD44 is regulated by various stimuli and metalloproteinases, and that CD44 cleavage plays a role in CD44-dependent transcriptional activity and cell migration. Recent studies by this group have focused on the important role of ADAM17-mediated cleavage of CD44 in oral SCC metastasis and invasion [32, 33]. Others have also found an important relationship between invasive ulcerative colitis carcinoma and CD44 cleavage [60]. These studies are consistent with our findings on the important role of CD44 cleavage in promoting stemness and tumorigenesis.

In conclusion, our findings demonstrate that CD44 cleavage by ADAM17 promotes orasphere formation or stemness and HNSCC tumorigenesis. While monoclonal antibodies against specific CD44 isoforms have been tested in patients with HNSCC for potential imaging or targeting therapy against tumors [61], our data support the notion that therapeutic pharmaceuticals that target CD44 cleavage mechanisms within the stem cell compartment can impair stemness and thus hold promise for treating aggressive oral cancer.

## References

[1] Bsoul SA, Huber MA, Terezhalmy GT (2005). Squamous cell carcinoma of the oral tissues: a comprehensive review for oral healthcare providers. J. Contemp. Dent. Pract.

[2] Al-Hajj M, Wicha MS, Benito-Hernandez A, Morrison SJ, Clarke MF (2003). Prospective identification of tumorigenic breast cancer cells. Proc. Natl. Acad. Sci. USA.

[3] Singh SK, Hawkins C, Clarke ID (2004). Identification of human brain tumour initiating cells. Nature.

[4] Collins AT, Berry PA, Hyde C, Stower MJ, Maitland NJ (2005). Prospective identification of tumorigenic prostate cancer stem cells. Cancer Res.

[5] Prince ME, Sivanandan R, Kaczorowski A (2007). Identification of a subpopulation of cells with cancer stem cell properties in head and neck squamous cell carcinoma. Proc. Natl. Acad. Sci. USA.

[6] Kokko LL, Hurme S, Maula SM (2011). Significance of site-specific prognosis of cancer stem cell marker CD44 in head and neck squamous-cell carcinoma. Oral Oncol.

[7] Aruffo A, Stamenkovic I, Melnick M, Underhill CB, Seed B (1990). CD44 is the principal cell surface receptor for hyaluronate. Cell.

[8] Zoller M (2011). CD44: can a cancer-initiating cell profit from an abundantly expressed molecule?. Nat. Rev. Cancer.

[9] Li C, Heidt DG, Dalerba P (2007). Identification of pancreatic cancer stem cells. Cancer Res.

[10] Ahrens T, Assmann V, Fieber C (2001). CD44 is the principal mediator of hyaluronic-acid-induced melanoma cell proliferation. J. Invest. Dermatol.

[11] Morrison H, Sherman LS, Legg J (2001). The NF2 tumor suppressor gene product, merlin, mediates contact inhibition of growth through interactions with CD44. Genes Dev.

[12] Goebeler M, Kaufmann D, Brocker EB, Klein CE (1996). Migration of highly aggressive melanoma cells on hyaluronic acid is associated with functional changes, increased turnover and shedding of CD44 receptors. J. Cell Sci.

[13] Klingbeil P, Marhaba R, Jung T, Kirmse R, Ludwig T, Zoller M (2009). CD44 variant isoforms promote metastasis formation by a tumor cell-matrix cross-talk that supports adhesion and apoptosis resistance. Mol. Cancer Res.

[14] Wang SJ, Bourguignon LY (2011). Role of hyaluronan-mediated CD44 signaling in head and neck squamous cell carcinoma progression and chemoresistance. Am. J. Pathol.

[15] Harper LJ, Costea DE, Gammon L, Fazil B, Biddle A, Mackenzie IC (2010). Normal and malignant epithelial cells with stem-like properties have an extended G2 cell cycle phase that is associated with apoptotic resistance. BMC Cancer.

[16] Toole BP, Slomiany MG (2008). Hyaluronan: a constitutive regulator of chemoresistance and malignancy in cancer cells. Semin. Cancer Biol.

[17] Bates RC, Edwards NS, Burns GF, Fisher DE (2001). A CD44 survival pathway triggers chemoresistance via lyn kinase and phosphoinositide 3-kinase/Akt in colon carcinoma cells. Cancer Res.

[18] Shin SI, Freedman VH, Risser R, Pollack R (1975). Tumorigenicity of virus-transformed cells in nude mice is correlated specifically with anchorage independent growth in vitro. Proc. Natl. Acad. Sci. USA.

[19] Kantak SS, Kramer RH (1998). E-cadherin regulates anchorage-independent growth and survival in oral squamous cell carcinoma cells. J. Biol. Chem.

[20] Grimshaw MJ, Cooper L, Papazisis K (2008). Mammosphere culture of metastatic breast cancer cells enriches for tumorigenic breast cancer cells. Breast Cancer Res.

[21] Swan EA, Jasser SA, Holsinger FC, Doan D, Bucana C, Myers JN (2003). Acquisition of anoikis resistance is a critical step in the progression of oral tongue cancer. Oral Oncol.

[22] Kupferman ME, Patel V, Sriuranpong V (2007). Molecular analysis of anoikis resistance in oral cavity squamous cell carcinoma. Oral Oncol.

[23] Bunek J, Kamarajan P, Kapila YL (2011). Anoikis mediators in oral squamous cell carcinoma. Oral Dis.

[24] Zhang Y, Lu H, Dazin P, Kapila Y (2004). Squamous cell carcinoma cell aggregates escape suspension-induced, p53-mediated anoikis: fibronectin and integrin alphav mediate survival signals through focal adhesion kinase. J. Biol. Chem.

[25] Kamarajan P, Alhazzazi TY, Danciu T (2012). D'Silva N J, Verdin E, Kapila YL. Receptor-interacting protein (RIP) and Sirtuin-3 (SIRT3) are on opposite sides of anoikis and tumorigenesis. Cancer.

[26] Ponti D, Costa A, Zaffaroni N (2005). Isolation and in vitro propagation of tumorigenic breast cancer cells with stem/progenitor cell properties. Cancer Res.

[27] Costea DE, Tsinkalovsky O, Vintermyr OK, Johannessen AC, Mackenzie IC (2006). Cancer stem cells – new and potentially important targets for the therapy of oral squamous cell carcinoma. Oral Dis.

[28] Chiou SH, Yu CC, Huang CY (2008). Positive correlations of Oct-4 and Nanog in oral cancer stem-like cells and high-grade oral squamous cell carcinoma. Clin. Cancer Res.

[29] Chen SF, Chang YC, Nieh S, Liu CL, Yang CY, Lin YS (2013). Nonadhesive culture system as a model of rapid sphere formation with cancer stem cell properties. PLoS ONE.

[30] Nagano O, Murakami D, Hartmann D (2004). Cell-matrix interaction via CD44 is independently regulated by different metalloproteinases activated in response to extracellular Ca(2+) influx and PKC activation. J. Cell Biol.

[31] Nagano O, Saya H (2004). Mechanism and biological significance of CD44 cleavage. Cancer Sci.

[32] Takamune Y, Ikebe T, Nagano O (2007). ADAM-17 associated with CD44 cleavage and metastasis in oral squamous cell carcinoma. Virchows Arch.

[33] Takamune Y, Ikebe T, Nagano O, Shinohara M (2008). Involvement of NF-kappaB-mediated maturation of ADAM-17 in the invasion of oral squamous cell carcinoma. Biochem. Biophys. Res. Commun.

[34] Henson B, Li F, Coatney DD (2007). An orthotopic floor-of-mouth model for locoregional growth and spread of human squamous cell carcinoma. J. Oral Pathol. Med.

[35] Alhazzazi TY, Kamarajan P, Joo N (2011). Sirtuin-3 (SIRT3), a novel potential therapeutic target for oral cancer. Cancer.

[36] Dontu G, Wicha MS (2005). Survival of mammary stem cells in suspension culture: implications for stem cell biology and neoplasia. J. Mammary Gland Biol. Neoplasia.

[37] Freedman VH, Shin SI (1974). Cellular tumorigenicity in nude mice: correlation with cell growth in semi-solid medium. Cell.

[38] Eble JA, Haier J (2006). Integrins in cancer treatment. Curr. Cancer Drug Targets.

[39] Tang MK, Zhou HY, Yam JW, Wong AS (2010). c-Met overexpression contributes to the acquired apoptotic resistance of nonadherent ovarian cancer cells through a cross talk mediated by phosphatidylinositol 3-kinase and extracellular signal-regulated kinase 1/2. Neoplasia.

[40] Howe EN, Cochrane DR, Richer JK (2011). Targets of miR-200c mediate suppression of cell motility and anoikis resistance. Breast Cancer Res.

[41] Shanmugathasan M, Jothy S (2000). Apoptosis, anoikis and their relevance to the pathobiology of colon cancer. Pathol. Int.

[42] Kamarajan P, Kapila YL (2007). An altered fibronectin matrix induces anoikis of human squamous cell carcinoma cells by suppressing integrin alpha v levels and phosphorylation of FAK and ERK. Apoptosis.

[43] Chiarugi P (2008). From anchorage dependent proliferation to survival: lessons from redox signalling. IUBMB Life.

[44] Neiva KG, Zhang Z, Miyazawa M, Warner KA, Karl E, Nor JE (2009). Cross talk initiated by endothelial cells enhances migration and inhibits anoikis of squamous cell carcinoma cells through STAT3/Akt/ERK signaling. Neoplasia.

[45] Ishida H, Wada K, Masuda T (2007). Critical role of estrogen receptor on anoikis and invasion of squamous cell carcinoma. Cancer Sci.

[46] Ponta H, Sherman L, Herrlich PA (2003). CD44: from adhesion molecules to signalling regulators. Nat. Rev. Mol. Cell Biol.

[47] Hauptschein RS, Sloan KE, Torella C (2005). Functional proteomic screen identifies a modulating role for CD44 in death receptor-mediated apoptosis. Cancer Res.

[48] Hao JL, Cozzi PJ, Khatri A, Power CA, Li Y (2010). CD147/EMMPRIN and CD44 are potential therapeutic targets for metastatic prostate cancer. Curr. Cancer Drug Targets.

[49] To K, Fotovati A, Reipas KM (2010). Y-box binding protein-1 induces the expression of CD44 and CD49f leading to enhanced self-renewal, mammosphere growth, and drug resistance. Cancer Res.

[50] Maula SM, Luukkaa M, Grenman R, Jackson D, Jalkanen S, Ristamaki R (2003). Intratumoral lymphatics are essential for the metastatic spread and prognosis in squamous cell carcinomas of the head and neck region. Cancer Res.

[51] Lin JT, Chang TH, Chang CS (2010). Prognostic value of pretreatment CD44 mRNA in peripheral blood of patients with locally advanced head and neck cancer. Oral Oncol.

[52] Han J, Kioi M, Chu WS, Kasperbauer JL, Strome SE, Puri RK (2009). Identification of potential therapeutic targets in human head & neck squamous cell carcinoma. Head Neck Oncol.

[53] Tu LC, Foltz G, Lin E, Hood L, Tian Q (2009). Targeting stem cells-clinical implications for cancer therapy. Curr. Stem Cell Res. Ther.

[54] Li F, Tiede B, Massague J, Kang Y (2007). Beyond tumorigenesis: cancer stem cells in metastasis. Cell Res.

[55] Okamoto A, Chikamatsu K, Sakakura K, Hatsushika K, Takahashi G, Masuyama K (2009). Expansion and characterization of cancer stem-like cells in squamous cell carcinoma of the head and neck. Oral Oncol.

[56] Biddle A, Liang X, Gammon L (2011). Cancer Stem Cells in Squamous Cell Carcinoma Switch between Two Distinct Phenotypes That Are Preferentially Migratory or Proliferative. Cancer Res.

[57] Ailles L, Prince M (2009). Cancer stem cells in head and neck squamous cell carcinoma. Methods Mol. Biol.

[58] Yu CC, Tsai LL, Wang ML (2013). miR145 targets the SOX9/ADAM17 axis to inhibit tumor initiating cells and IL-6-mediated paracrine effects in head and neck cancer. Cancer Res.

[59] Zheng X, Jiang F, Katakowski M, Zhang ZG, Lu QE, Chopp M (2009). ADAM17 promotes breast cancer cell malignant phenotype through EGFR-PI3K-AKT activation. Cancer Biol. Ther.

[60] Mikami T, Yoshida T, Numata Y (2011). Invasive behavior of ulcerative colitis-associated carcinoma is related to reduced expression of CD44 extracellular domain: comparison with sporadic colon carcinoma. Diagn. Pathol.

[61] Lyons AJ, Jones J (2007). Cell adhesion molecules, the extracellular matrix and oral squamous carcinoma. Int. J. Oral Maxillofac. Surg.

